# The Promotion and Optimization of Bank Financial Products Using Consumers’ Psychological Perception

**DOI:** 10.3389/fpsyg.2022.926271

**Published:** 2022-07-25

**Authors:** Jing Zhang, Bo Jin

**Affiliations:** ^1^College of Finance, Shanxi University of Finance and Economics, Taiyuan, China; ^2^Department of Finance and Economics, Taiyuan University, Taiyuan, China

**Keywords:** consumer psychological perception, bank, financial products, marketing, strategy optimization

## Abstract

With the rapid economic growth and increased national income year by year, individuals and families have an increasingly greater demand for financial products. Banks’ sales of financial products have become a new economic profit growth point for major banks. Based on consumers’ psychological perception, the influencing factors of consumers’ behavior in purchasing bank financial products are studied. The influencing factor model path of consumer purchase behavior is constructed to find out the factors affecting consumers’ purchase of bank financial products and formulate appropriate promotion strategies according to the influencing factors. Through the research methods of literature analysis, small-scale in-depth interview, questionnaire surveys, and statistical analysis, this exploration selects four variables: independent variable, mediator, control variable, and dependent variable. They are influencing factors of purchasing bank financial products (perceived convenience, risk value of bank financial products, satisfaction of purchasing communication process), consumers’ willingness to buy bank financial products, consumers’ personal characteristics and consumers’ behavior of purchasing bank financial products. Meanwhile, based on 196 valid questionnaires, regression analysis is carried out through a regression model. The results show that the three influencing factors of consumers’ purchase of bank financial products–perceived convenience, risk value of bank financial products, and satisfaction with the purchase communication process significantly impact consumers’ purchase of bank financial products. They can put forward specific promotion suggestions for banks. This exploration aims to study the optimization of bank financial product promotion strategy from the perspective of consumer psychological perception to provide a reference for subsequent related research.

## Introduction

This exploration aims to improve the current promotion rate of bank financial products. China’s banking industry has developed rapidly in the past decade and a service network has been established all over the country. The number of users increases exponentially, forming a situation dominated by the four major state-owned commercial banks, supplemented by urban commercial banks, and foreign banks gradually enter. The competition is quite fierce (Tandukar et al., 2021). The financial needs of individuals and families are increasingly higher with the rapid economic growth and the increase of national income year by year. The prospect of bank retail business is bright, which has become a new economic profit growth point for major banks and attracted much attention. Bank retail service is a service activity provided to individuals, families, self-employed operators, and small and medium-sized enterprises with financial products as the carrier and customer demand as the guide (Haralayya, 2021). Therefore, whether financial products can meet customers’ needs and whether the quality of financial products can satisfy customers will greatly impact the development of banks (Wu et al., 2021). Most of China’s banks have experienced joint-stock reform and gradually embarked on the road of marketization. The formulation of development strategies is no longer with the planned economy as the guidance but with customers as the core. Banks can remain invincible in the fierce competition only by firmly grasping customer resources (Lubis et al., 2021).

Meanwhile, the serious homogenization of financial products, backward service concept and level, non-standard market competition and other problems have also seriously restricted the development of the domestic banking industry. Therefore, in the fierce competitive environment, the key factor of bank development is to change the service concept (Ferm and Thaichon, 2021). Banks should innovate financial products with customers as the core and pay attention to the psychological perceived value of consumers. Only by letting consumers perceive the value of services can they make purchase decisions (Chen, 2020). Western countries researched bank consumer perception earlier and have formed a perfect system. They have shifted the research focus to improving banking services (Algarni et al., 2021). The research on consumer perception of Bank of China was carried out late and did not form a system. Therefore, exploring the influencing factors of Bank of China consumers’ perceived value, gradually establishing a service process based on consumers’ psychological perceived value, allowing consumers to experience pleasant and satisfactory service marketing strategies in the service process, and increasing the opportunity of repeat purchase have become an important topic in China’s current bank marketing research (Prastiwi and Fitria, 2021). Based on the model of traditional economics, as long as the cost is equal to the income, it is in a balanced state. As long as financial institutions judge that the cost of a certain behavior is less than the benefit, they think that the behavior can be selected (Hsu et al., 2022). This assumption is wrong because the costs and benefits measured in money appear in the form of intervals. People will instinctively create various “frameworks” to produce a certain psychological perception, namely “psychological account.” The intervention of psychological account makes the cost and benefit can no longer be simply equivalent. The psychological account is an important aspect of people’s self-control. The example of financial self-control can be easily seen by observing some daily behaviors. For example, people often use separate budget accounts for decision-making. Part of the income is prepared for the decoration of the house. Some are used as funds for children’s education. Others are reserved for future pension or disease. Others pay for mortgage loans or daily consumption. This kind of expense is essentially the external manifestation of people’s “psychological account.” The idea behind people’s “psychological account” is that people usually do not treat money in the same way, and money cannot be replaced at will as envisaged by traditional economics. Therefore, in-depth analysis of consumers’ psychological perception is crucial for promoting bank financial products.

In China’s current fierce competition situation, how to promote bank financial products and obtain more bank internal capital based on consumers’ psychological perceptions has become one of the crucial issues concerned by banks (Shukla et al., 2022). Given the important influence of consumers’ psychological perception on the sales of bank financial products, it is necessary to investigate the role of bank financial products promotion under consumers’ psychological perception to explore more diversified bank financial products promotion strategies and provide reference opinions for improving the promotion of bank financial products. The research innovation is to study the influencing factors of consumers’ behavior of purchasing bank financial products from the perspective of consumers’ psychological perception, and put forward improvement strategies. First, consumers’ psychological perception is analyzed theoretically, and the research hypothesis is put forward. Next, a conceptual model is proposed and a questionnaire is designed. Finally, the questionnaire data analysis and correlation analysis are carried out. This exploration provides a reference for the effective promotion of bank financial products.

## Literature Review

Consumer psychological perception is mainly reflected in consumer satisfaction, so the literature review part mainly analyzes and synthesizes the literature related to consumer satisfaction. The concept of customer satisfaction was first put forward in 1965. It is defined as that customer satisfaction with products or services will increase the tendency of customers to repeat purchases, and then improve the market share and profit margin of enterprises (Lee and Lee, 2022). Hou and Sarigöllü (2022) defined the overall service satisfaction as a function of all service quality, which was the degree to which consumers like or dislike the service after going through the purchase process. Therefore, customer satisfaction is an overall attitude based on experience (Hou and Sarigöllü, 2022). Liu et al. (2022) believed that satisfaction was the overall attitude of customers towards the products or services used (Liu et al., 2022). Shimul et al. (2022) believed that satisfaction was a personal subjective feeling expressed by measuring the perceived utility of products or services and consumers’ expectations (Shimul et al., 2022).

The research on customer satisfaction in China is carried out in combination with the consumption characteristics of Chinese customers. Scholars have conducted a lot of research on satisfaction and other influencing factors and put forward unique views (Pittman et al., 2022). Lin and Shi (2022) proposed that customer expectations positively and negatively affected satisfaction, but the positive impact was generally the main impact (Lin and Shi, 2022). Zhang M. et al. (2022) proposed that customer perceived value is an important prerequisite for determining customer satisfaction. There is an interaction between customer perceived value and customer satisfaction at different levels, thus forming different levels of customer satisfaction (Zhang M. et al., 2022). The definition of satisfaction is also controversial. However, generally, the measurement of satisfaction comes from the difference between customers’ expected value and customers’ perceived value in consumption. Dissatisfaction will appear when the perceived value does not meet the expected value. When the value felt by customers is equal to or even exceeds customer expectations, customers will be satisfied or even very satisfied. Zhang J. et al. (2022) proposed a service profit chain model that expounded the relationship between customer value and satisfaction. Customer satisfaction is determined by the value perceived by customers (Zhang J. et al., 2022). Customer perceived value will play a more crucial role in operation and management in a typical service industry. Zhang (2021) demonstrated this hypothesis and concluded that perceived value positively impacted customer satisfaction (Zhang, 2021). Yang et al. (2021) believed that customer perceived value positively impacted customer satisfaction and customer repeat purchase intention (Yang et al., 2021).

Previous research on consumer satisfaction mostly focused on the theoretical level, which was too general. Besides, there were few studies on consumer psychological perception: satisfaction under the promotion of bank financial products. Therefore, this exploration takes the actual situation of banks as the research object to explore the relationship between consumer satisfaction and banking services. It has strong practicability.

## Theoretical Basis and Research Hypothesis

### Consumer Psychological Perception

**FIGURE 1:**
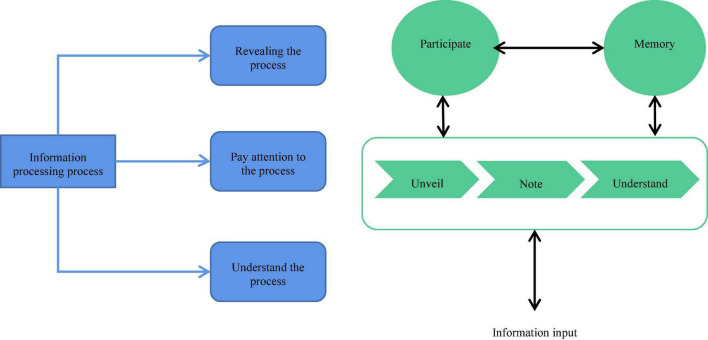
Flow chart of information’s cognitive processing. **(A)** Stage diagram of information processing. **(B)** Flow chart of information processing.

Consumer behavior is defined as what people do when they get what they use. In the consumer behavior study, it is necessary to analyze the psychology and behavior of different consumers and the factors affecting consumer behavior (Kim, 2021). The two-factor emotion model divides customer perception psychology into positive and negative aspects (Prahiawan et al., 2022). Consumer’s psychological perception is divided into consumers’ feelings and perceptions. In daily life, consumers always face various information and are attracted by some of it. They process the information to get understanding and judgment, make decisions and take actions. The process of consumers’ processing of external information is also the process of consumers’ understanding and cognition of consumption objects (Shareef et al., 2021). From the perspective of psychology, the cognitive process of information is the core part of consumers’ psychological process. Figure 1 shows the cognitive processing process of consumers on information.

Figure 1 reveals that the consumer information cognitive processing process is divided into three stages. (1) Reveal the process: consumers feel external information through their own sensory organs. In this stage, the sensory theory of psychology plays a leading role. (2) Pay attention to the process: the attention principle of psychology explains how consumers configure their information processing ability among massive information. (3) Understand the process: consumers sort out, process, and analyze the information they focus on to form consumer perception (Fletcher-Brown et al., 2021). The analysis reveals that the most important influencing factor of the consumer feeling stage is the degree of consumer participation. Consumer participation refers to a state of motivation generated by consumers’ awareness of the importance of consumer products or interest caused by external stimuli. With the deepening of participation, consumers have a stronger motivation to contact, pay attention to and understand consumer products (Peters and Bodkin, 2022). In particular, in the information understanding stage, the deeper the consumer participation is, the more comprehensive and deeper their understanding of consumer product information is. Then, it will improve the consumer’s willingness to consume. Hence, understanding the consumer participation degree positively affects product promotion (Mattison Thompson and Brouthers, 2021). The degree of consumer participation also determines the nature of their purchase decision to some extent. For consumers with high participation, their decision-making process goes through stages of demand awareness, information search, evaluation scheme, purchase decision, and post-purchase measurement, which belongs to extensive decision-making behavior. Low participation consumers have limited time and energy invested in purchase activities. The purchase decision-making process is simple, which belongs to limited decision-making purchase behavior (de Oliveira et al., 2022).

Consumers’ perception of information reflects their perception of information stimulus intensity and its changes. The appropriate stimulus range of information can be calculated by sensitivity and threshold (Okojie and Stankievech, 2021). Among them, absolute sensitivity refers to the ability to detect the minimum amount of stimulation. The absolute threshold is the minimum value of information stimulus that can make people feel (Feeney et al., 2021). Equation (1) is the relationship between absolute sensitivity and absolute threshold:


(1) $$\text{E}=\frac{1}{\text{R}}$$

*E* is absolute receptivity; *R* is the absolute threshold.

The difference perception threshold refers to the minimum difference between two pieces of information that consumers can perceive (Lalouni et al., 2021). Difference sensitivity refers to the minimum sensitivity to differential sensory threshold (Chen and Liu, 2021). Equation (2) is the relationship between sensory intensity and stimulus intensity:


(2) S=Kl⁢o⁢g⁢B+C

*S* is the sensory intensity; *B* is the stimulation intensity; *K* and *C* are constants.

In practical application, marketers can use the threshold to calculate the product improvement scope to avoid repeated waste of resources. Alternatively, they can use the psychological threshold of consumers to adjust the factors affecting consumers’ purchase intention and keep the influencing factors within an appropriate range to maintain consumers’ purchase intention (Hasan M. et al., 2021).

### Proposal of Research Hypotheses and Conceptual Model

According to the relevant literature on consumer behavior, academic research on the promotion of bank financial products mainly focuses on consumers’ personal characteristics, characteristic factors of financial products, and consumers’ perceived risk (Erlangga and Erlangga, 2021). The influencing factor analysis of bank financial products promotion here is also from these three perspectives to analyze their impact on consumers’ purchase of bank financial products.

The in-depth interview method is adopted to investigate the influencing factors affecting the promotion of bank financial products. Consumers’ motivation, attitude, and emotion to buy bank financial products are investigated. The survey data show that the usefulness and convenience of consumers’ purchase of financial products will affect consumers’ willingness to buy the bank’s financial products. In the technology acceptance model, perceived convenience is proposed. It is defined as that compared with the traditional way of selecting bank financial products, the purchase process is simple and consumers are willing to understand (Binder et al., 2021). Consumers can understand various information about financial products through the network platform. Relevant studies believe that the higher the perceived convenience is, the stronger the consumer’s purchase intention is (Keni et al., 2022). Therefore, this exploration takes the perceived convenience as an influencing factor for consumers to buy bank financial products. Accordingly, the hypothesis below is put forward:

H1: Perceived convenience has a significant positive impact on consumers’ willingness to buy bank financial products.

During the survey, respondents hold that the risk of buying financial products will impact consumers’ purchase intention. Before purchasing financial products, consumers will first consider the comparison value of the return and risk of financial products (Tran et al., 2022). Therefore, this exploration takes financial products’ risk value as an influencing factor for consumers to buy bank financial products. Hewei and Youngsook (2022) showed that the low risk of financial products would positively impact consumers’ purchase of bank financial products (Hewei and Youngsook, 2022). Accordingly, the following hypothesis is put forward:

H2: The risk value of financial products significantly impacts consumers’ willingness to buy bank financial products.

Meanwhile, respondents believe that appropriate communication methods and reception processes will affect consumers’ emotions in the purchase process, thus affecting their purchase intention. Therefore, the communication process is regarded as an influencing factor for consumers to buy bank financial products. Arslanagic-Kalajdzic et al. (2022) found that the high-quality service of bank service personnel and active communication with consumers positively impacted consumers’ final purchase of financial products (Arslanagic-Kalajdzic et al., 2022). Accordingly, the following hypothesis is put forward:

H3: The communication process has a significant positive impact on consumers’ willingness to buy bank financial products.

The previous research literature review reveals that purchase intention is the subjective probability or possibility of consumers buying a specific product. The stronger the consumers’ intention is, the greater the possibility of implementation behavior is (Ong et al., 2022). Based on this, the following hypothesis is put forward:

H4: Consumers’ willingness to buy bank financial products has a significant positive impact on the behavior of buying bank financial products (Felix et al., 2022).

Different consumer characteristics will also cause the respondents’ different perceptions of buying bank financial products in the survey. In the theory of planned behavior, it is also believed that the behavior intention is affected by the individuals themselves. It is embodied in that the individual’s personality, intelligence, age, and gender will indirectly affect the behavioral intention and behavior (Klaus and Zaichkowsky, 2022). Moreover, in the technology acceptance model, it is also pointed out that perceived convenience will also be affected by individual characteristics (Wang et al., 2021). Therefore, consumer characteristics are set as control variables to consider their impact on other variables. The consumer characteristics here mainly include the gender, age, educational level, and monthly income of consumers. Based on this, the following hypotheses are put forward:

H5a: Different consumer characteristics significantly impact the influencing factors of consumers’ purchase of bank financial products.

H5b: Different consumer characteristics significantly impact consumers’ willingness to buy bank financial products.

H5c: Different consumer characteristics significantly impact consumers’ behavior of buying bank financial products.

### Questionnaire and Data Analysis

#### Questionnaire Design

**FIGURE 2:**
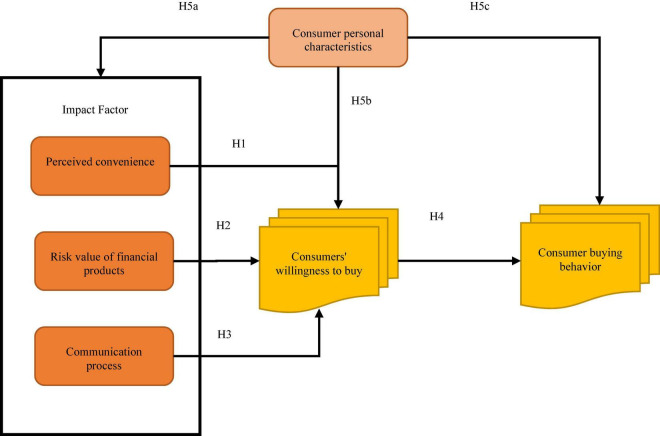
Model diagram of the study.

Likert’s 5-point scale is used to measure variables. Consumers’ willingness to buy bank financial products is used as a mediator, and consumers’ characteristics are used as a control variable to study the impact of influencing factors on consumers’ behavior of buying bank financial products (Lei and Zhang, 2021). Based on the relevant research literature, this exploration will focus on the influencing factors of consumers’ purchase of bank financial products (Siddiqi et al., 2022). A research model on the influencing factors of consumers’ purchase of bank financial products based on psychological perception is proposed. Figure 2 shows the specific research model.

According to the research model diagram, the evaluation items of the questionnaire are designed. This questionnaire is divided into two parts. The first part is to investigate the basic information of the participants, including gender, age, education level, and annual income. The second part is consumers’ perceived satisfaction with the communication services of bank financial products, consumers’ evaluation of the risk value of financial products and consumers’ evaluation of the convenience of purchasing bank financial products. This part includes 20 questions transformed from the proposed research conceptual model indexes. Likert’s 5-point scale evaluation method is adopted (Uzun et al., 2022), making the respondents fill in the degree of agreement with the items. Five representatives “agree very much,” three representatives “generally,” and one representative “disagrees.”

#### Descriptive Analysis

The questionnaire method is adopted to collect research data and investigate consumers who buy bank financial products. In total, 200 questionnaires were distributed and 196 valid questionnaires were recovered, with an effective recovery rate of 98%. Statistical Product Service Solutions (SPSS) statistical software is used for data analysis. Table 1 displays the sample data obtained.

**TABLE 1:** Sample datasheet.

Sample data		Number of people	Percentage
Gender	Male	105	53.57%
	Female	91	46.43%
Age	≤25 years old	70	35.71%
	26–35 years old	65	33.16%
	≥36 years old	61	31.13%
Educational level	Junior college	42	21.42%
	Undergraduate	78	39.79%
	Master degree or above	76	38.79%
Monthly income	<3,000	45	22.95%
	3,000–5,000	68	34.69%
	5,000<	83	42.36%

Cronbach α coefficient is adopted to evaluate the survey results to determine the stability and reliability of the survey results (Hasan M. K. et al., 2021). The calculation equation of Cronbach α coefficient is:


(3) α=n⁢r/[(n-1)⁢r+1]

*n* is the scale’s item quantity, and *r* is the average correlation coefficient between items. By comparing the Cronbach α coefficient with the data reliability requirements, whether the data meet the experimental reliability requirements is judged (Caycho-Rodríguez et al., 2022).

Kaiser-Meyer-Olkin (KMO) sample measure and Bartlett’s test are conducted on the effective sample data by factor analysis to judge the correlation among the selected variables (Venugopal et al., 2022).

## Research Results and Analysis

### Reliability and Validity Analysis of the Scale

**FIGURE 3:**
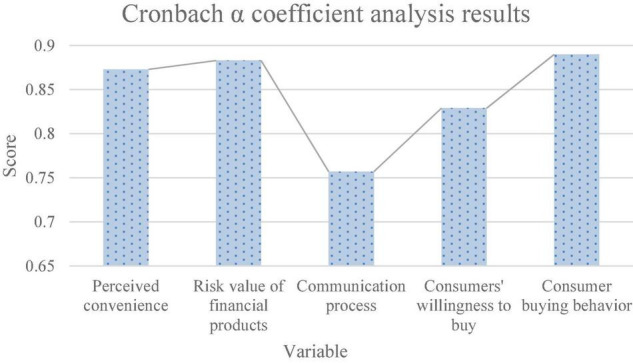
Analysis results of the Cronbach α coefficient.

Figure 3 shows the analysis results of the Cronbach α coefficient.

Figure 3 reveals that the Cronbach α coefficient score of perceived convenience is 0.873. The risk value of bank financial products is 0.883. The score of the communication process is 0.757. The score of consumers’ willingness to buy bank financial products is 0.829. Consumers’ behavior of purchasing bank financial products scores 0.890. The Cronbach α coefficient scores of variables are greater than the standard scores, which meets the requirements for data reliability analysis, indicating that the collected measurement data have high reliability and stability.

**FIGURE 4:**
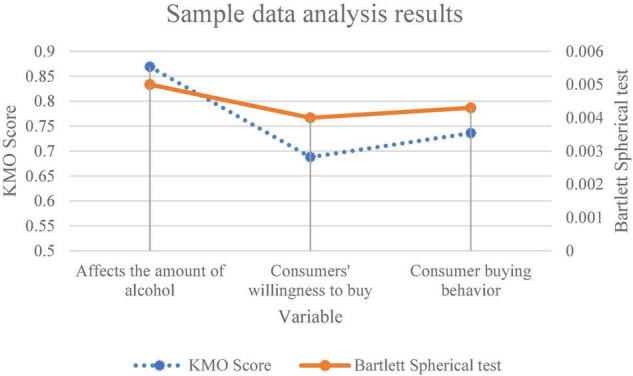
Analysis results of sample data.

Figure 4 shows the results of factor analysis of sample data.

Figure 4 reveals that the KMO values of the scale of influencing factors of consumers’ behavior in purchasing bank financial products, the scale of consumers’ willingness to purchase bank financial products and the scale of consumers’ behavior in purchasing bank financial products are 0.869, 0.688, and 0.736, respectively. Their significance probabilities of Bartlett’s test are 0.005, 0.004, and 0.0043, which are less than the significance standard of 0.01 and in line with the general research standard of statistics for the effectiveness of the scale. Therefore, the survey data can be used for this exploration.

### Analysis on the Relationship Between Consumer Purchase Influencing Factors and Consumer Purchase Intention

**FIGURE 5:**
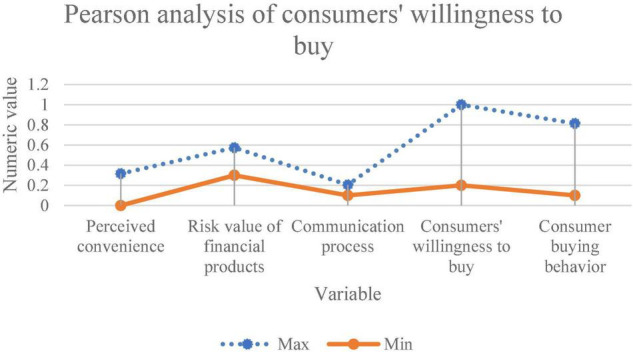
Correlation analysis between influencing factors of consumer purchase and consumer purchase intention.

The correlation between the influencing factors of consumers’ purchase of bank financial products and consumers’ willingness to buy bank financial products is analyzed. Figure 5 displays the analysis results.

Figure 5 suggests that the significant coefficient between the influencing factors of consumers’ purchase of bank financial products and consumers’ willingness to purchase bank financial products is *p* = 0.0085 < 0.01, indicating a strong correlation. Moreover, there is a significant positive correlation between them. In the latest research, Rahmi et al. (2022) revealed that consumers’ purchase intention directly affected purchase behavior, which was basically consistent with the conclusion here. On this basis, this exploration conducts more detailed research on bank financial products (Rahmi et al., 2022). Further, regression analysis between the influencing factors of consumers’ purchase of bank financial products and consumers’ willingness to purchase bank financial products is made. Table 2 shows the results of the regression analysis.

**TABLE 2:** The results of the regression analysis.

Variable	Perceived convenience	Risk value of financial products	Communication process	Sig.F	Coefficient of determination	Adjust coefficient of determination
Score	0.316	0.574	0.205	0.324	0.642	0.464

In Table 2, Sig.F = 0.324 suggests a good fitting degree between the influencing factors of consumer purchase and the variables of consumer purchase intention. The coefficient of determination is 0.642, indicating that the influencing factors of consumers’ purchase of bank financial products can explain the 64.2% difference of consumers’ willingness to purchase bank financial products. The fitting degree of the two is high, suitable for regression analysis, which indicates an obvious linear relationship between them. Meanwhile, it confirms the conclusion of a significant correlation between the two in the previous section. Therefore, it can be concluded that the hypotheses H1, H2, and H3 are true. It means that perceived convenience, risk value of financial products, and communication process significantly impact consumers’ purchase intention. Research on consumers’ psychological perceptions has proved this. As mentioned above, a lower risk of financial products will increase consumers’ purchase intention. Buenafe et al. (2022) also confirmed that the quality of communication affects consumers’ purchase intention. On this basis, this exploration concludes that consumers’ purchase intention is significantly related to the communication process (Buenafe et al., 2022).

### Analysis on the Relationship Between Consumers’ Purchase Intention and Consumers’ Purchase Behavior

The correlation between consumers’ willingness to buy bank financial products and consumers’ behavior of buying bank financial products is analyzed. Table 3 shows the analysis results.

**TABLE 3:** Results of correlation analysis.

	Pearson correlation	Sig.(2-tailed)	*N*
Consumer purchase intention	0.282	0.004	196
Consumer purchase behavior	0.387	0.001	196

**FIGURE 6:**
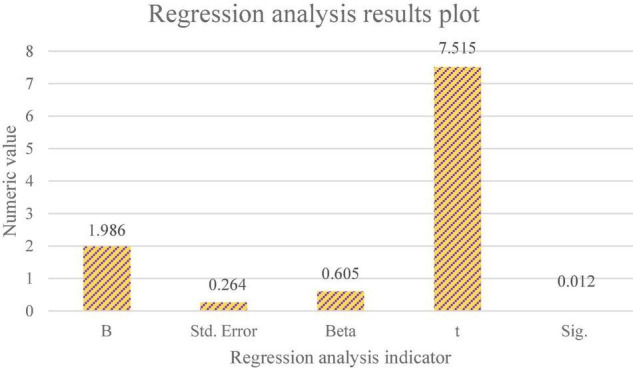
Regression analysis results of consumers’ willingness to buy products and consumers’ behavior of buying products. B in the figure is the value of the regression coefficient. Std. Error is the standard error. Beta is the standardized regression coefficient value. The value of t is the calculated intermediate value. Sig. is the significance index value.

When the significance level is 0.01 and Sig. value is less than or equal to 0.01, the possibility of no significant correlation between the two variables is less than 0.01. Table 2 reveals a significant positive correlation between consumers’ willingness to buy bank financial products and consumers’ behavior to buy bank financial products. Further, a regression analysis between consumers’ willingness to buy bank financial products and consumers’ behavior to buy bank financial products is made. Figure 6 shows the results.

Figure 6 shows that the regression coefficient of consumers’ willingness to buy products is 1.986, the t statistic value is 7.515, and the *p*-value is 0.000. Therefore, the regression coefficient of the explanatory variable conforms to the significance test, that is, there is a significant linear relationship between consumers’ willingness to buy bank financial products and consumers’ behavior of buying bank financial products. Besides, the Sig. value of t statistic of the constant term is 0.012 < 0.05, which also conforms to the significance test. It again proves the significant positive impact between consumers’ willingness to buy bank financial products and consumers’ behavior to buy bank financial products. Therefore, H4 is true. The research of Frommeyer et al. (2022) showed that there was a significant correlation between intention and behavior, which was consistent with the research conclusion here (Frommeyer et al., 2022).

### Analysis on the Relationship Between Consumer Characteristics and Consumer Purchase Intention, Behavior, and Influencing Factors

The correlation between consumers’ personal characteristics and consumers’ willingness, behavior and influencing factors to buy bank financial products is analyzed. Table 4 shows the analysis results.

**TABLE 4:** Results of correlation analysis between consumers’ personal characteristics and purchase intention, behavior, and influencing factors.

		Purchase intention	Purchase behavior	Influencing factors of purchase
Gender	Pearson correlation	0.355	0.269	0.090
	Sig.(2-tailed)	0	0.007	0.381
	*N*	196	196	196
Age	Pearson correlation	0.218	–0.174	0.284
	Sig.(2-tailed)	0.01	0.081	0.004
	*N*	196	196	196
Educational level	Pearson correlation	0.316	0.574	0.251
	Sig.(2-tailed)	0.013	0.017	0.028
	*N*	196	196	196
Monthly income	Pearson correlation	0.387	0.306	0.241
	Sig.(2-tailed)	0.023	0.014	0.005
	*N*	196	196	196

**FIGURE 7:**
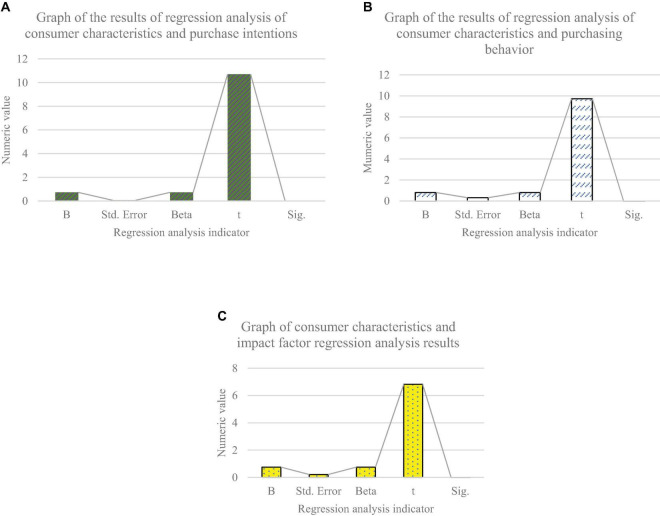
Regression analysis results of consumer characteristics and consumer purchase intention, behavior, and influencing factors. **(A)** Analysis results of consumer characteristics and purchase intention. **(B)** Analysis results of consumer characteristics and purchase behavior. **(C)** Analysis results of consumer characteristics and influencing factors of purchase intention.

Table 4 shows that the Sig. values of the variables are basically less than 0.05, so there is a significant relationship between the variables. It means that consumers’ personal characteristics have a significant positive correlation with consumers’ willingness to buy bank financial products, purchase behavior and purchase influencing factors. Further regression analysis is made on the consumers’ personal characteristics and their willingness, behavior and influencing factors to buy bank financial products. Figure 7 shows the results.

Figure 7 shows that the regression coefficients of consumers’ willingness to buy products are 0.735, 0.814, and 0.756, and the t-statistics are 10.719, 9.732, and 6.831. Therefore, the regression coefficients of explanatory variables comply with the significance test, that is, there is a weak linear relationship between consumer characteristics and consumers’ willingness, behavior and influencing factors to buy products. Besides, the Sig. values of t statistics of constant terms are less than 0.05, which also conforms to the significance test. It once again proves that consumer characteristics have a significant positive impact on consumers’ willingness, behavior and influencing factors to buy products. Therefore, H5a, H5b, and H5c are true. The research of Sharma et al. (2022) on consumer characteristics showed that consumer characteristics significantly affected consumer behavior, which was consistent with the results here. On this basis, this exploration obtains a more specific impact relationship (Sharma et al., 2022).

## Conclusion

Through theoretical and investigation research, this exploration focuses on the influencing factors of consumers’ purchase of bank financial products based on consumers’ psychological perception, and obtains the following main conclusions. The three influencing factors of consumers’ purchase of bank financial products–perceived convenience, risk value of bank financial products and satisfaction with the communication process have a significant impact on consumers’ purchase of bank financial products. Therefore, banks can recommend and reform bank financial products from these three aspects. The risk value of bank financial products is the main influencing factor for consumers to buy bank financial products, followed by perceived convenience and satisfaction with the communication process. It shows the important considerations of consumers for purchasing bank financial products, and provides a critical optimization path for enterprises to promote bank financial products. The research results are basically consistent with the current research results on consumers’ psychological perception. This exploration puts forward the following suggestions. First, it is necessary to stimulate consumers’ interest in bank financial products. Based on consumers’ interest in bank financial products, banks should optimize the publicity path of bank financial products, and provide different publicity paths according to different consumer characteristics. Then, the quality of communication with consumers should be ensured. Banks should focus on consumers’ feelings, and maintain consumers’ interest in bank financial products. Next, they should gradually improve bank financial products, launch comprehensive and stable financial products that meet the requirements of consumers, and reduce consumers’ risk perception. The research deficiency is that there are few influencing factors, and consumers may be affected by more aspects in real life. Follow up research needs to consider more factors affecting consumers’ purchase intention. The practical research value is that it is expected to be applied to the services of state-owned commercial banks, reduce consumers’ perceived cost and increase consumers’ purchase intention.

## Data Availability Statement

The original contributions presented in this study are included in the article/supplementary material, further inquiries can be directed to the corresponding author.

## Ethics Statement

Ethical review and approval was not required for the study on human participants in accordance with the local legislation and institutional requirements. Written informed consent from the patients/participants or patients/participants legal guardian/next of kin was not required to participate in this study in accordance with the national legislation and the institutional requirements.

## Author Contributions

Both authors listed have made a substantial, direct, and intellectual contribution to the work, and approved it for publication.

## Conflict of Interest

The authors declare that the research was conducted in the absence of any commercial or financial relationships that could be construed as a potential conflict of interest.

## Publisher’s Note

All claims expressed in this article are solely those of the authors and do not necessarily represent those of their affiliated organizations, or those of the publisher, the editors and the reviewers. Any product that may be evaluated in this article, or claim that may be made by its manufacturer, is not guaranteed or endorsed by the publisher.
